# Microbiological Profile of Chronic Tonsillitis in the Pediatric Age Group

**DOI:** 10.7759/cureus.3343

**Published:** 2018-09-22

**Authors:** Raja Kalaiarasi, Kalaivani S Subramanian, Chellappa Vijayakumar, Ramakrishnan Venkataramanan

**Affiliations:** 1 Otorhinolaryngology, Sri Lakshmi Narayana Institute of Medical Science, Puducherry, IND; 2 Pathology, Sri Manakula Vinayagar Medical College, Puducherry, IND; 3 Surgery, Jawaharlal Institute of Postgraduate Medical Education and Research, Puducherry, IND; 4 Otolaryngology, Sri Lakshmi Narayana Institute of Medical Science, Puducherry, IND

**Keywords:** tonsils, tonsillectomy, bacteria, actinomyces

## Abstract

Introduction

Tonsillitis is a very common disease in children. Understanding the microbiology and pathology of chronic tonsillitis is an important step in its management. The aim of the study was to describe the microbiological profile of core tonsillar tissue in chronic tonsillitis in children.

Materials and methods

Children under 16 years of age with chronic tonsillitis were recruited in the descriptive study. Children with recurrent tonsillitis and recurrent tonsillitis with obstructive symptoms were included. Children who underwent tonsillectomy for obstructive symptoms alone and those who received antibiotics for at least one month prior to surgery were excluded from the study. Dissection and the snare method of tonsillectomy were done on all children. The operated specimen was cut into two halves in a sterile container. The core of the tonsillar tissue was swabbed with two sterile cotton-tipped swabs and sent for the microbiological evaluation of aerobes and anaerobes. The tonsillar tissue was sent for a histopathological examination.

Results

A total of 106 children were operated for chronic tonsillitis in one year. The mean age of children included in this study was 9.4 years. The duration of symptoms due to tonsillar disease ranged from four weeks to 28 months. There were 48 males and 58 females. Recurrent tonsillitis was the most common indication for tonsillectomy in all children. A total of 301 aerobes and 171 anaerobic microorganisms were isolated from 106 children with chronic tonsillitis. The aerobic bacterial species most often isolated was Streptococcus viridans, which was present in 83 children followed by Group A, β-hemolytic Streptococci in 67 children. The anaerobic bacterial most often isolated was Peptococcus species in 49 children. Polymicrobial aerobic and anaerobic flora were present in all tonsillar specimens, yielding an average of 4.1 isolates per specimen. The histopathological examination revealed chronic tonsillitis with reactive follicular hyperplasia in all (100%) children. Actinomycosis was associated with non-specific reactive follicular hyperplasia in four specimens.

Conclusion

Polymicrobial aerobic and anaerobic flora are identified in deep tonsillar tissue in children with tonsillitis. The identification of bacterial isolates from the core tissue in recurrent tonsillitis could dictate the management of chronic tonsillitis. The histopathological examination of the core tissues of the tonsils helps in an accurate identification of organisms that are difficult to culture.

## Introduction

Tonsils are subepithelial lymphoid tissue in the oropharynx between the palatoglossal pillar anteriorly and the palatopharyngeal pillar posteriorly. Tonsils are in a region where microorganisms are found in ample. Microorganisms penetrate into the tonsillar tissue through the defect in the epithelium and get access to the lymphatic system, which is responsible for all the individual attacks of tonsillitis [1]. Hence, it is important to know the individual organism causing tonsillitis.

Tonsillitis is a very common disease in children. Tonsillectomy is the most common surgical procedure performed in children with recurrent tonsillitis. The greatest immunological activity of the tonsil is found between the ages of three to 10 years. As a result, the tonsils are more prominent during this period and later demonstrate age-dependent involution [2]. One or more attacks of acute tonsillitis per year are common in the primary school age group children. The common symptoms include sore throat, dysphagia, and fever with or without a history of upper respiratory tract infection. Children experiencing recurrent tonsillitis may develop enlarged tonsillar crypts with debris, persistent congestion of the tonsils, and dilated blood vessels on the surface of tonsils. Tonsillar diseases affect other anatomical-related structures like the middle ear cleft, paranasal sinuses, and upper aerodigestive tract [2-3]. In chronic tonsillitis, a culture of organisms obtained from the tonsillar surface might not be the infecting organism but could be the colonizing species. So, core culture from the tonsil would be more reliable. Thus, understanding the microbiology and pathology of chronic tonsillitis from the deep core tissue is an important step in its management. The aim of the descriptive study was to describe the microbiological profile of core tonsillar tissue in children with chronic tonsillitis.

## Materials and methods

This descriptive study included all children under 16 years of age with chronic tonsillitis presenting to the otorhinolaryngology outpatient department (OPD) for a one-year duration at a tertiary teaching center in South India. Institute ethical committee clearance was obtained for the study. Children were recruited for the study after explaining the nature, methodology, and risks involved to the parents or guardians. Participants were given full freedom to withdraw themselves from the study at any time.

Recurrent tonsillitis is described as more than seven episodes in one year, more than five episodes annually for more than two years, more than three episodes annually for more than three years, or two weeks or more of lost school in one year due to tonsillitis. The clinical features of sore throat plus the presence of temperature >38.3^0^C, cervical lymphadenopathy (tender lymph nodes or >2 cm), tonsillar exudates, or a positive culture for Group A beta-hemolytic Streptococcus [4-5]. Children with recurrent tonsillitis and recurrent tonsillitis with obstructive symptoms were included in the study. Children who underwent tonsillectomy for obstructive symptoms alone and children who received antibiotics at least one month prior to surgery were excluded from the study.

All children underwent a detailed history and a thorough ear, nose, and throat examination. Children fulfilling inclusion criteria were recruited for the study. Demographic details like age, sex, presenting symptoms, and tonsillar enlargement were studied. Dissection and the snare method of tonsillectomy were done on all children. The operated specimen was cut into two halves in a sterile container. The core of the tonsillar tissue was swabbed with two sterile cotton-tipped swabs and sent for a microbiological evaluation. In the microbiology laboratory, the swab was cultured on blood agar, MacConkeys agar for aerobic and facultative organisms, and on anaerobic medium for the culture of anaerobic organisms. The identification of the organisms was done using standard techniques [6-7]. The growth of the microbial colonies in the culture media was studied. The tonsillar tissue was sent for a histopathological examination. The tonsils were fixed in formalin and embedded in paraffin blocks from which microscopic slides were prepared. The slides were stained with Haemotoxylin and Eosin (H&E). All categorical variables were reported using percentages/proportions. All continuous variables were reported using mean and standard deviation or median and interquartile range. Statistical analysis was done using SPSS 19.0 software for Windows (IBM, Armonk, New York, United States). 

## Results

A total of 106 children were operated for chronic tonsillitis in one year. The mean age of children included in this study was 9.4 years. The duration of symptoms due to tonsillar disease ranged from four weeks to 28 months. There were 48 males and 58 females. Recurrent tonsillitis was the most common indication for tonsillectomy in all children. Totally, 106 tonsils were studied. A total of 301 aerobes and 171 anaerobic microorganisms were isolated from 106 children with chronic tonsillitis. The aerobic bacterial species most often isolated was Streptococcus viridans, which was present in 83 children followed by Group A, β-hemolytic Streptococci in 67 children (Table 1).

**Table 1:** Aerobic organisms studied in 106 excised tonsillar specimens from children with chronic tonsillitis

S.No	Aerobic isolates	No. (%)
1.	Streptococcus viridans	83 (27.57%)
2.	Group A, β- hemolytic Streptococci	67 (22.25%)
3.	Streptococcus pneumonae	42 (13.95%)
4.	Staphylococcus aureus	38 (12.62%)
5.	Haemophilus influenzae type B	21 (6.98%)
6.	Group B, β- hemolytic Streptococci	18 (5.98%)
7.	Diphtheroid species	18 (5.98%)
8.	Haemophilus species	5 (1.66%)
9.	Pseudomonas aeruginosa	3 (0.99%)
10.	Escherichia coli	2 (0.66%)
11.	Candidia albicans	2 (0.66%)
12.	Staphylococcus epidermidis	2 (0.66%)
	Total	301

The anaerobic bacterial most often isolated was Peptococcus species in 49 children (Table 2).

**Table 2:** Anaerobic organisms studied in 106 excised tonsillar specimens from children with chronic tonsillitis

S.No	Anaerobic isolates	No. (%)
1.	Peptococcus species	49 (28.65%)
2.	Bacteroides species	36 (21.05%)
3.	Fusobacterium species	31 (18.12%)
4.	Veillonella species	23 (13.45%)
5.	Lactobacillus species	18 (10.52%)
6.	Peptostreptococcus species	12 (7.02%)
7.	Actinomycetes species	2 (1.17%)
	Total	171

Polymicrobial aerobic and anaerobic flora were present in all tonsillar specimens, yielding an average of 4.1 isolates per specimen. No definite bacterial combinations were observed in the core tissue culture.

**Figure 1:**
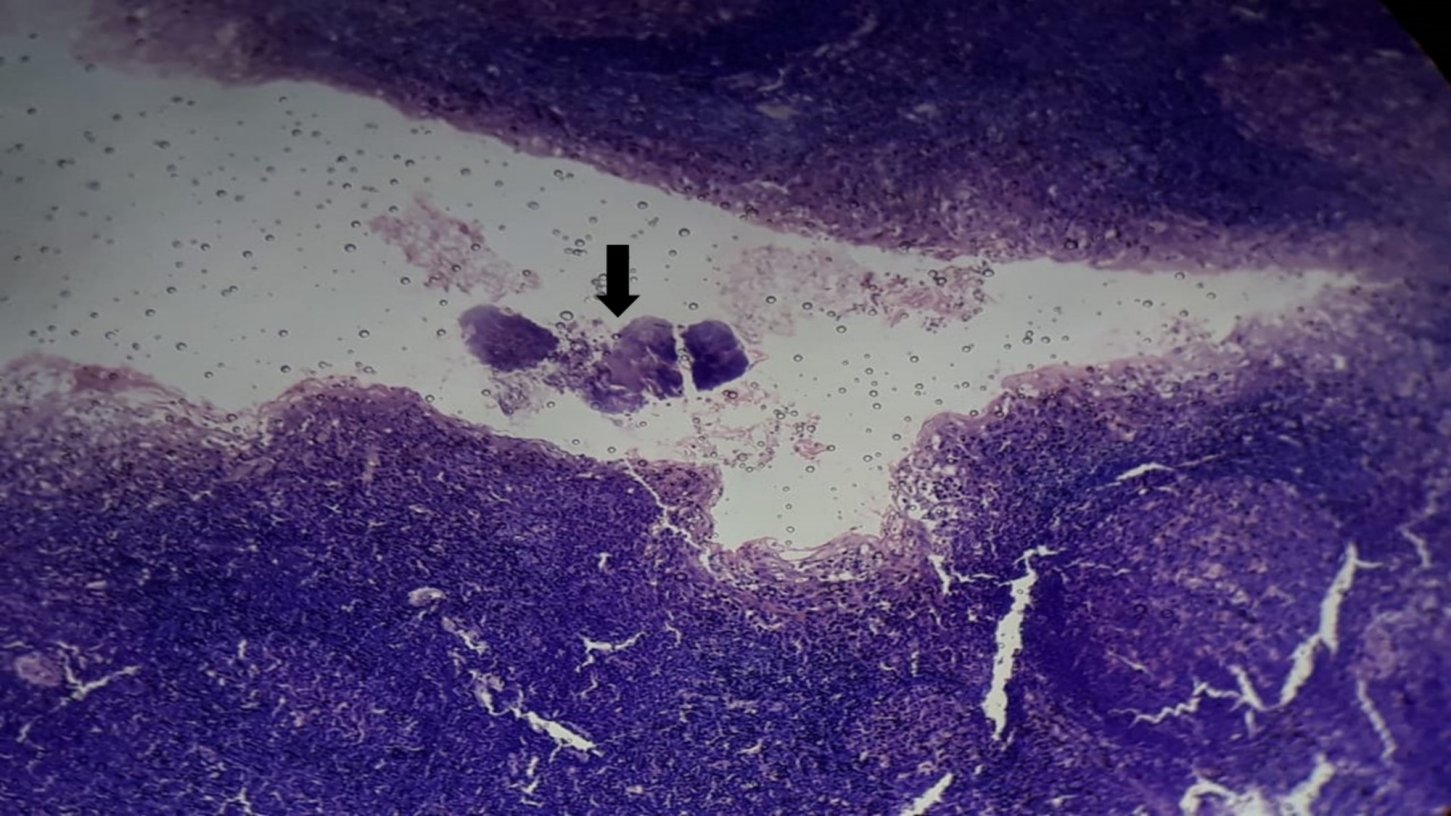
Histopathological examination showing the colonization of the tonsillar crypt (black arrow) by Actinomycotic colonies (Hematoxylin and Eosin, 100X)

The histopathological examination revealed chronic tonsillitis with reactive follicular hyperplasia in all (100%) children. Actinomycosis was associated with non-specific reactive follicular hyperplasia in four specimens. In three patients, the slide showed just the colonization of the tonsillar crypt by colonies of actinomyces (Figure 1).

**Figure 2:**
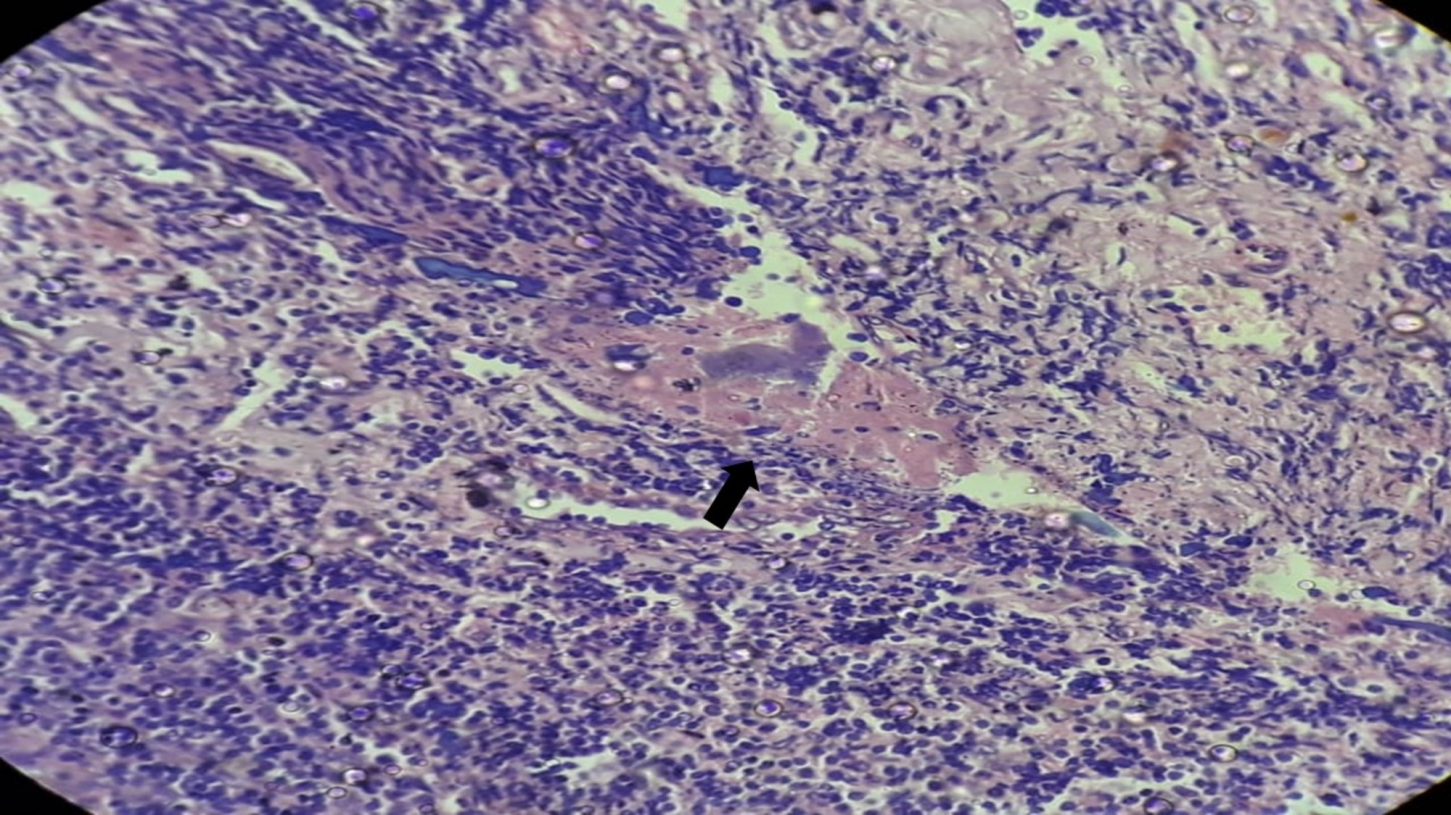
Histopathological examination showing tonsillar parenchymal infiltration by Actinomycotic colonies surrounded by the Splendore-Hoeppli phenomenon (black arrow) (Hematoxylin and Eosin, 400X)

Actinomyces are filamentous basophilic microorganisms arranged in a radial spoke-like fashion. In one specimen, there was the infiltration of organisms into tissue parenchyma with Splendore-Hoeppli reaction in addition to the colonization of crypts (Figure 2).

## Discussion

Most of the research work on the microbiological study of chronic tonsillitis was aimed at identifying the aerobic organisms. The role of anaerobes in chronic tonsillitis is rarely studied. Anaerobes are normally commensal in the oropharynx, so cultures taken from the surface may be misleading. In the present study, the role of both aerobes and anaerobes were studied. Cultures were taken from the deep tonsillar tissue to study the disease-causing organisms.

Common aerobic organisms isolated were Streptococcus viridans, group A, β-hemolytic Streptococci, Streptococcus pneumoniae, Staphylococcus aureus, Haemophilus influenzae type B, group B β-hemolytic Streptococci, and diphtheroid species. In the study by Agrawal et al. on all age group patients, common causative organisms isolated from the tonsillar surface were alpha-hemolytic Streptococci, Staphylococcus aureus, non-pathogenic Neisseria species, Haemophilus influenza, Pneumococcus, Enterococcus, Bacteroid fragilis, and Corynebacterium species. No anaerobes were identified [8]. In another study by Omer et al. in Turkey, an attempt was made to isolate the facultative and obligate anaerobes from the surface and core of patients with recurrent tonsillitis [9]. The common isolated facultative anaerobic species were coagulase-negative Staphylococci, alpha-hemolytic Streptococci, and Diphtheroid bacilli, whereas Neisseria species substituted Diphtheroid bacilli in children under eight years. On the other hand, the commonest isolated obligate anaerobic species were Propionibacterium acnes, Prevotella melaninogenica, and Peptostreptococcus anaerobius.

In this study, common anaerobes isolated were Peptococcus species, Bacteroid species, Fusobacterium species, Veillonella species, Lactobacillus species, and Peptostreptococcus species. No common pattern of a combination of aerobes and anaerobes were studied in the tonsillar specimen. Actinomyces were isolated in two specimens in culture. The histopathological examination of the specimen showed non-specific reactive follicular hyperplasia in all (100%) children secondary to infection. Actinomycosis was associated with follicular hyperplasia in four specimens of which one specimen showed actinomycotic infiltration of the tonsillar parenchymatous tissue. This indicates actinomyces are etiological microorganism causing tonsillitis.

Actinomycosis is commonly reported in the craniofacial, pulmonary, and ileocecal areas. It is also reported in the liver, breast, parotid, and spleen [10]. Tonsillar actinomycosis has been reported in a variable percent in the literature [11-12]. Tonsillar actinomycosis may indicate an etiological role in tonsillar hypertrophy and recurrent tonsillitis [13]. In their study, Gaffney et al. found no correlation between tonsillitis and actinomycosis and concluded actinomyces are saprophytes in the tonsillar tissue [12]. Kansu et al. identified a higher rate of cryptitis with actinomycosis and concluded actinomyces are pathogenic organisms in tonsillitis [14]. In this study, we found colonization and tissue infiltration. Tissue infiltration and the presence of the Splendore-Hoeppli phenomenon are indicators of tonsillitis due to actinomyces. Actinomyces colonization in the crypts and tissue infiltration can be differentiated only by H&E staining [15]. The Splendore-Hoeppli phenomenon (asteroid bodies) is the formation of eosinophilic material (asteroid shaped configurations) around actinomyces [16]. Tonsillar actinomycosis requires a long-term high dose penicillin treatment to eradicate the organism even after tonsillectomy surgery when compared to other infectious etiology. The limitations of the study include small sample size and short duration. Further randomized trials with a larger sample size and studying the antibiotic sensitivity pattern of the microorganisms may provide added evidence.

## Conclusions

The identification of bacterial isolates from the core tissue in recurrent tonsillitis could dictate the management of chronic tonsillitis. A histopathological examination of the core tissues of the tonsils helps in the accurate identification of organisms that are difficult to culture. The histological examination also helps to differentiate colonization from tissue infiltration.
